# Supplementary figures and images for: Migraine and gastroesophageal reflux disease: Disentangling the complex connection with depression as a mediator
Source: PLoS One. 2024 Jul 11;19(7):e0304370. doi: 10.1371/journal.pone.0304370 (PMC11239078; doi:10.1371/journal.pone.0304370)

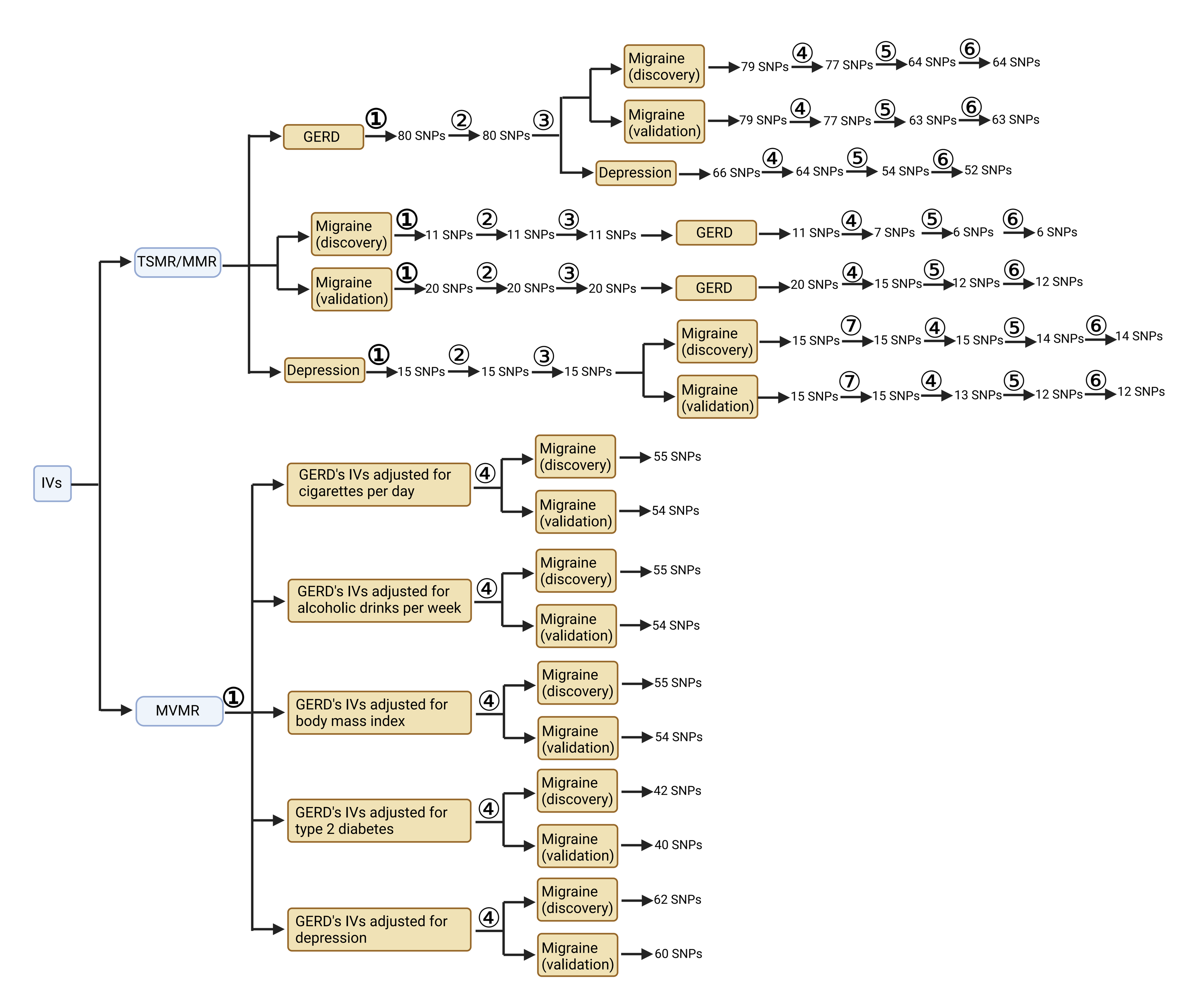

Supplement: S1 Fig — (TIF) [file pone.0304370.s001.tif]

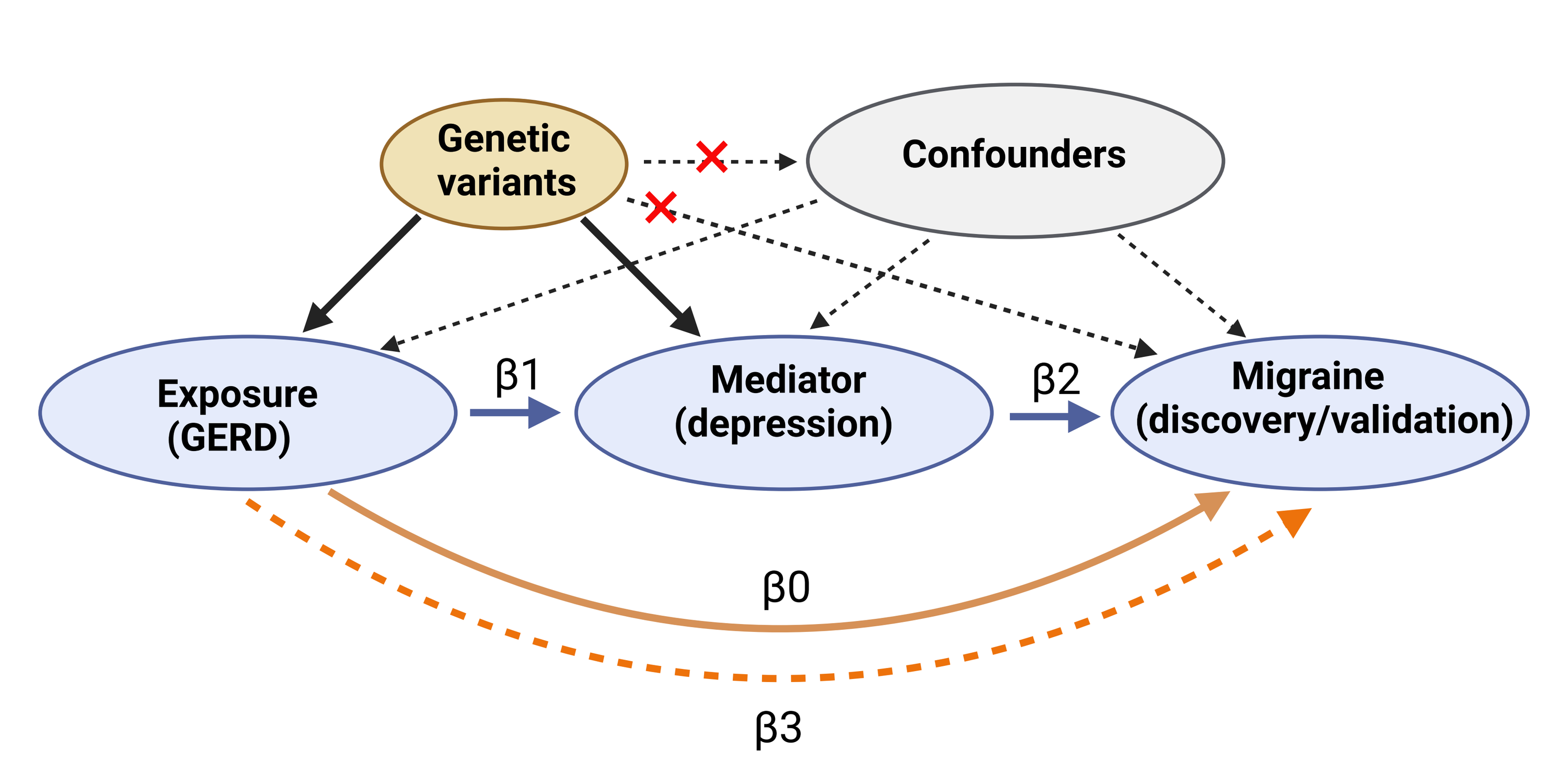

Supplement: S2 Fig — (TIF) [file pone.0304370.s002.tif]

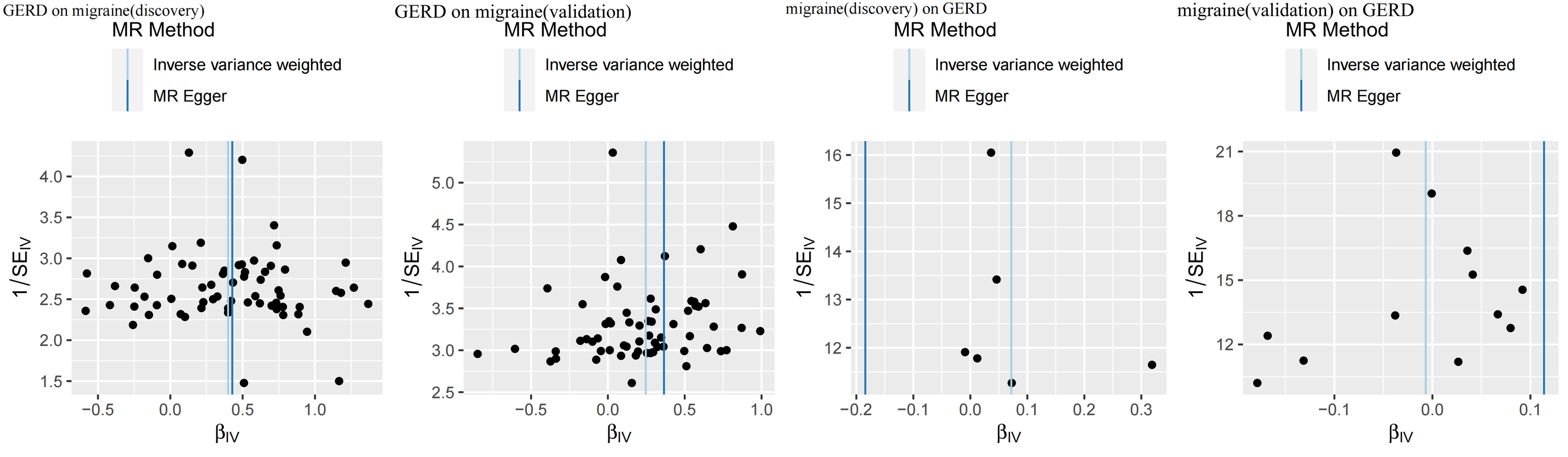

Supplement: S3 Fig — (TIF) [file pone.0304370.s003.tif]

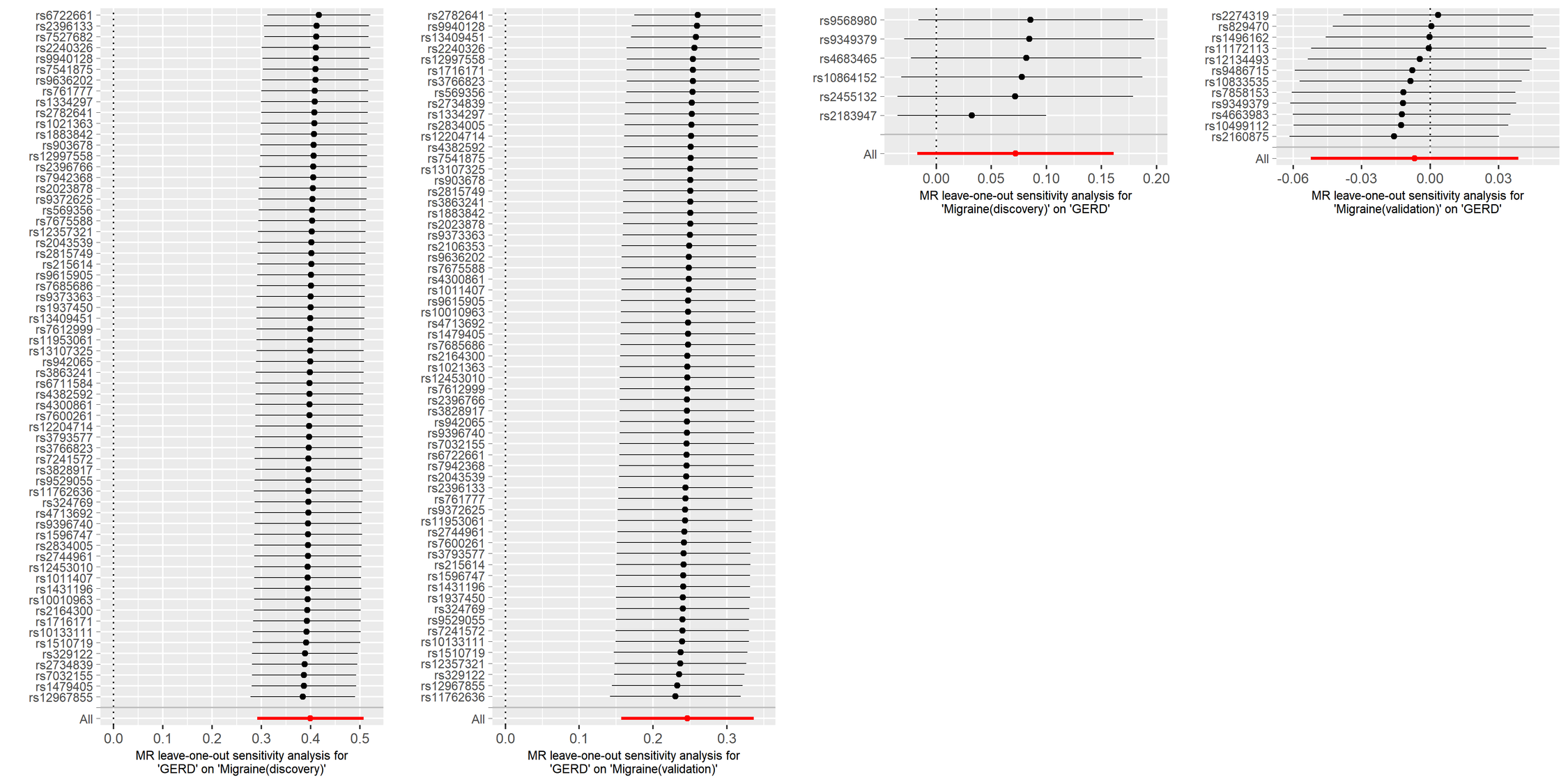

Supplement: S4 Fig — (TIF) [file pone.0304370.s004.tif]

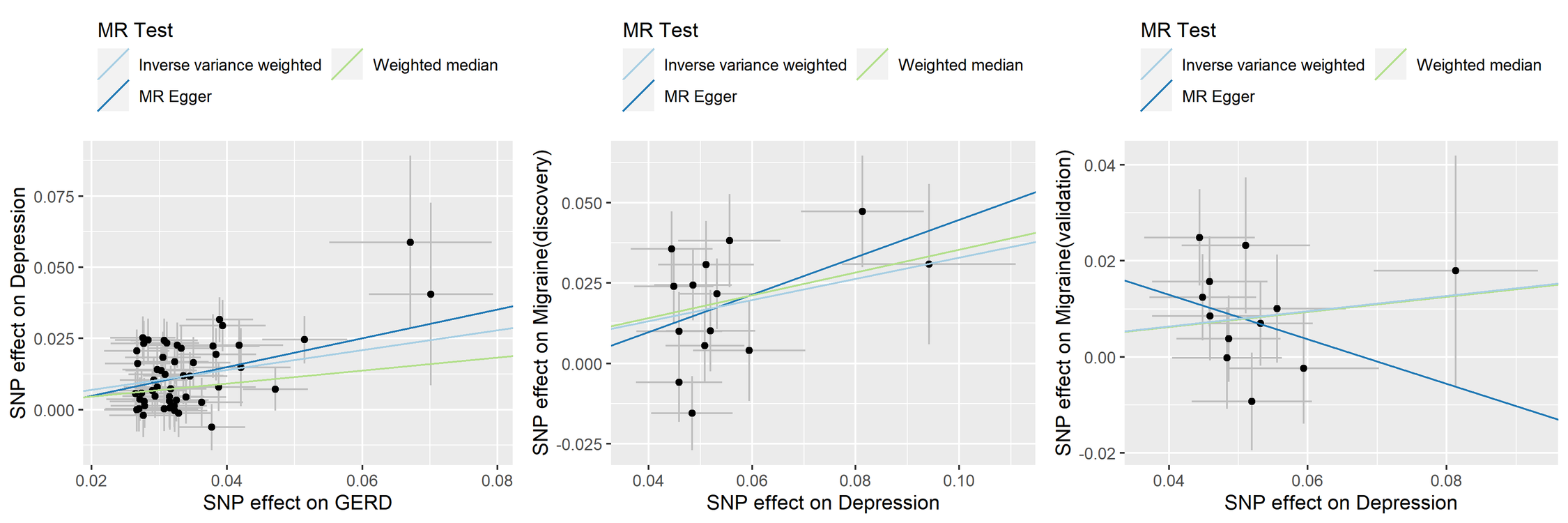

Supplement: S5 Fig — (TIF) [file pone.0304370.s005.tif]

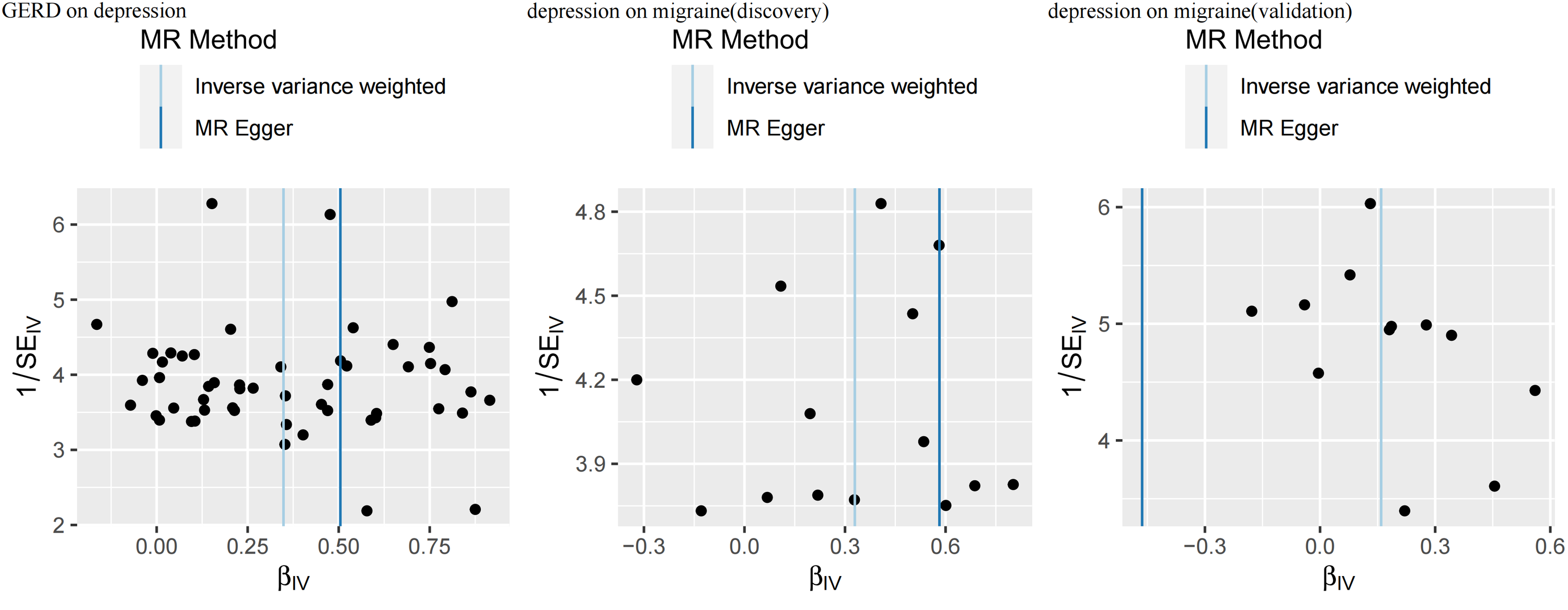

Supplement: S6 Fig — (TIF) [file pone.0304370.s006.tif]

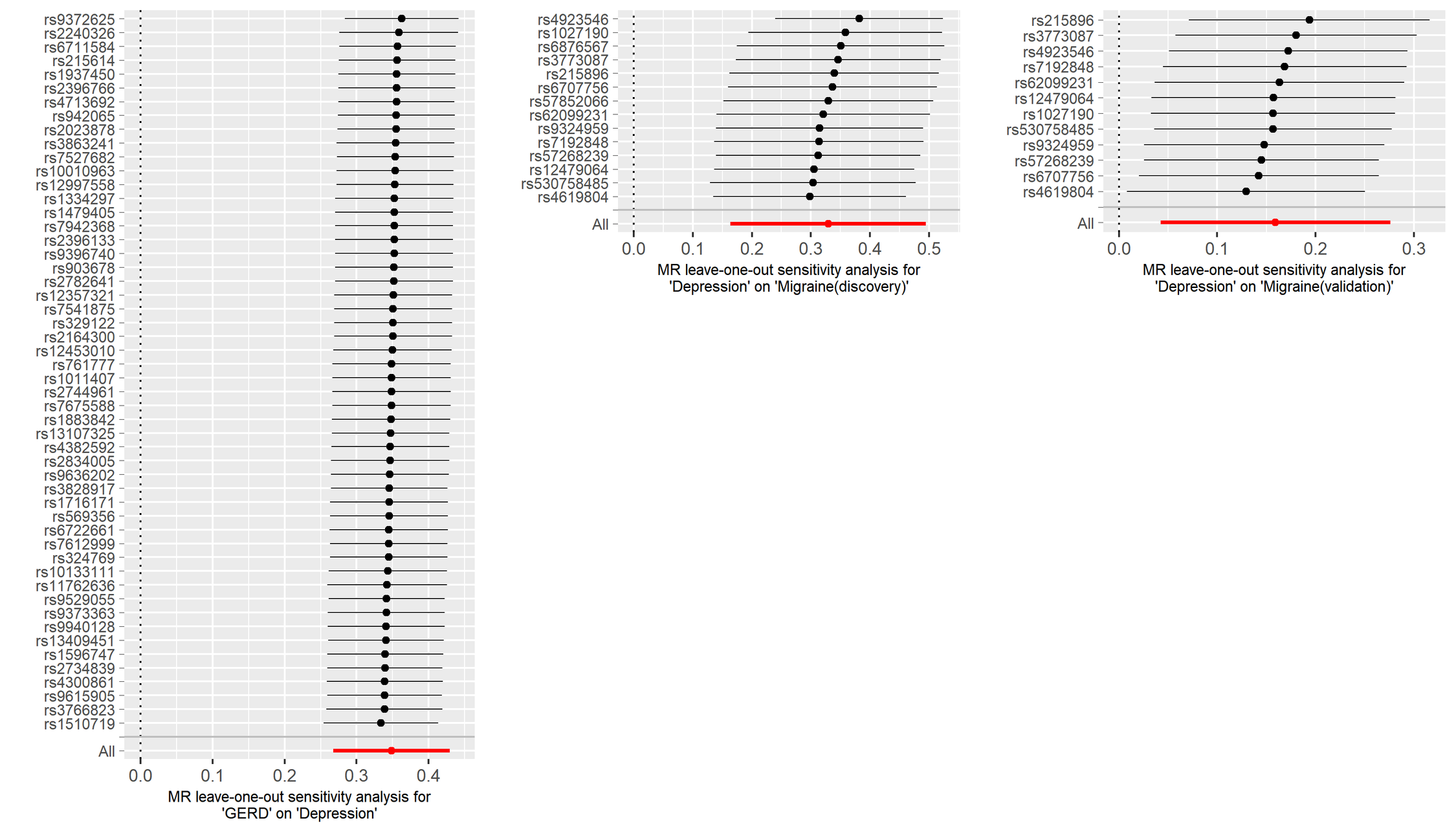

Supplement: S7 Fig — (TIF) [file pone.0304370.s007.tif]

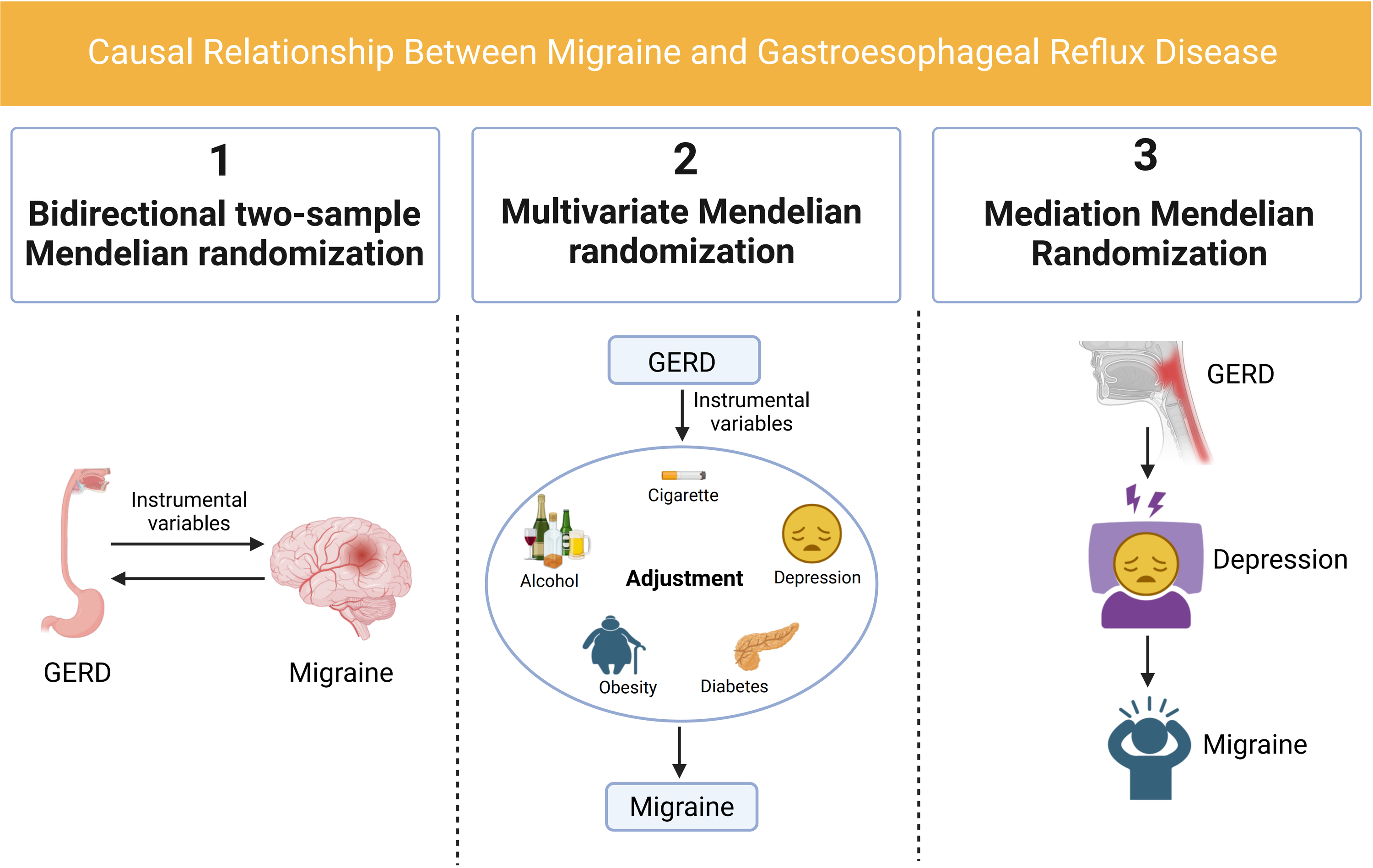

Supplement: S1 Graphical abstract — (TIF) [file pone.0304370.s009.tif]
