# Supplementary material for: Migraine and gastroesophageal reflux disease: Disentangling the complex connection with depression as a mediator
Source: PLoS One. 2024 Jul 11;19(7):e0304370. doi: 10.1371/journal.pone.0304370 (PMC11239078; doi:10.1371/journal.pone.0304370)
Supplement: Attachment — Submitted filename: Response to Reviewers.docx [file pone.0304370.s010.docx]

**Response to academic editor**

Dear Editor, thank you for giving us the opportunity to revise our manuscript. We have carefully read all your comments as well as those of the reviewers. Subsequently, we have made thorough and comprehensive revisions to our manuscript. We hope that our modifications meet the high standards of both you, the reviewers, and the journal. If you have any further comments, we would be more than willing to engage in further discussion. Below are our responses to the comments from you and the reviewers:

1.If applicable, we recommend that you deposit your laboratory protocols in protocols.io to enhance the reproducibility of your results. Protocols.io assigns your protocol its own identifier (DOI) so that it can be cited independently in the future. For instructions see: https://journals.plos.org/plosone/s/submission-guidelines#loc-laboratory-protocols. Additionally, PLOS ONE offers an option for publishing peer-reviewed Lab Protocol articles, which describe protocols hosted on protocols.io. Read more information on sharing protocols at https://plos.org/protocols?utm_medium=editorial-email&utm_source=authorletters&utm_campaign=protocols.

**Response:** Dear Editor, Our study is based on genome-wide association studies from existing public databases. Therefore, storing experimental protocols on Protocols.io seems inappropriate for our research. However, to ensure clarity for other readers regarding the methodology of our study, we have provided detailed descriptions of the entire study design and all data sources in the manuscript. This will enable other researchers to replicate our study smoothly based on our explanations.

2. Please ensure that your manuscript meets PLOS ONE's style requirements, including those for file naming. The PLOS ONE style templates can be found at

https://journals.plos.org/plosone/s/file?id=wjVg/PLOSOne_formatting_sample_main_body.pdf and

https://journals.plos.org/plosone/s/file?id=ba62/PLOSOne_formatting_sample_title_authors_affiliations.pdf

**Response:** We have carefully reviewed the style requirements of PLOS One and have made comprehensive and meticulous revisions to the manuscript.

3.We suggest you thoroughly copyedit your manuscript for language usage, spelling, and grammar. If you do not know anyone who can help you do this, you may wish to consider employing a professional scientific editing service.

Whilst you may use any professional scientific editing service of your choice, PLOS has partnered with both American Journal Experts (AJE) and Editage to provide discounted services to PLOS authors. Both organizations have experience helping authors meet PLOS guidelines and can provide language editing, translation, manuscript formatting, and figure formatting to ensure your manuscript meets our submission guidelines. To take advantage of our partnership with AJE, visit the AJE website (http://aje.com/go/plos) for a 15% discount off AJE services. To take advantage of our partnership with Editage, visit the Editage website (www.editage.com) and enter referral code PLOSEDIT for a 15% discount off Editage services. If the PLOS editorial team finds any language issues in text that either AJE or Editage has edited, the service provider will re-edit the text for free.

Upon resubmission, please provide the following:

The name of the colleague or the details of the professional service that edited your manuscript

A copy of your manuscript showing your changes by either highlighting them or using track changes (uploaded as a *supporting information* file)

A clean copy of the edited manuscript (uploaded as the new *manuscript* file)

**Response:** Dear editor, we have carefully read all the review comments from you and the three reviewers, and have done our best to make detailed revisions to the manuscript. And the manuscript has been marked accordingly according to your comments. We hope that our revisions can meet the high standards of you and the reviewers. If you and the reviewers have more comments, we will be happy to communicate with you further. We used the professional language editing service from Bullet Edits in the UK and uploaded the service certification documents.

4.Thank you for stating in your Funding Statement:

This study was supported by The Key Research and Development Plan (Major Science and Technology Special Project) of the Sichuan Provincial Science and Technology Department (No.2022YFS0392) and the Key Discipline Construction Project of the Sichuan Provincial Administration of Traditional Chinese Medicine (No.202072).

Please provide an amended statement that declares all the funding or sources of support (whether external or internal to your organization) received during this study, as detailed online in our guide for authors at http://journals.plos.org/plosone/s/submit-now. Please also include the statement “There was no additional external funding received for this study.” in your updated Funding Statement.

Please include your amended Funding Statement within your cover letter. We will change the online submission form on your behalf.

**Response:** Dear Editor, We have refined our funding information in accordance with the requirements of PLOS One and described it in the cover letter.

5.Please include captions for your Supporting Information files at the end of your manuscript, and update any in-text citations to match accordingly. Please see our Supporting Information guidelines for more information: http://journals.plos.org/plosone/s/supporting-information.

**Response:** We have added supporting information to the manuscript.

**Response to Reviewer #1**

Dear Reviewer #1, thank you for your valuable suggestions to improve our manuscript. We have improved the manuscript based on your comments. Below are responses to your comments.

1.In Table 1, the definitions of various phenotypes used in the GWAS data should have a more detailed range based on the International Classification of Diseases.

**Response:** Dear Reviewer, we have provided detailed supplemental definitions for the phenotypes used in the genome-wide association study. You can refer to Table 1 in our latest submission for clarification.

2.If possible, I suggest the authors include leave-one-out results, funnel plots, and scatter plots for the mediation Mendelian randomization analysis in the supplementary methods section.

**Response:** Dear reviewer, we have incorporated leave-one-out, funnel plots, and scatter plots results involved in the mediation Mendelian randomization analysis into the supplementary methods. We appreciate your meticulous requirements for our manuscript, which have contributed to its improvement.

**Response to Reviewer #2**

Dear Reviewer #2, we sincerely appreciate your valuable feedback and admire your rigorous scientific spirit. We have carefully reviewed all of your comments and made meticulous revisions to our manuscript. It is evident that the quality of our paper has significantly improved, thanks to your insightful suggestions for modification. Through this revision process, we have gained valuable expertise in manuscript writing, which we will continue to apply and refine in our future work. Below is our detailed response to your feedback, and we hope our revisions meet your high standards. Please do not hesitate to contact us if you have any further review comments.

1. I recommend revising the title. Please present a title that conveys the most important message of this manuscript. Suggestions: "Unraveling the Causal Relationship Between Migraine and Gastroesophageal Reflux Disease: A Mendelian Randomization Study"; "Migraine and GERD: Disentangling the Complex Connection with Depression as a Mediator" [1 –3].

**Response:** After careful consideration of the characteristics of our study, we have chosen the second manuscript title recommended by you.

2. A graphical abstract that visually summarizes the main findings of this manuscript is highly recommended.

**Response:** We have created a graphical abstract based on our research findings. You can find it in the latest manuscript.

3. Abstract: I would like the authors to make as much effort in this section for the remainder of the manuscript. Please expand the abstract in 200 words (preferably 200–220 words; max. 250), although the journal allows 300 words [4], focusing on proportionally presenting the background, methods, results, and conclusion (without the headings of the subsections). The background should include the general background (one to two sentences), the specific background (two to three sentences), and "the current issue addressed to this study" (one sentence), leading to the objectives. Additionally, the methods should clarify the authors’ approach, such as the study design and variables, to solve the problem and/or make progress on the problem. The results should be close to a single sentence, thus putting the results in a more general context. The conclusion should open with one sentence describing the main result using such words as “Here we show,” which should be followed by statements such as the potential and the advance this study has provided in the field, and finally a broader perspective (two to three sentences) readily comprehensible to a scientist in any discipline [5–8].

**Response:** Thank you for your valuable suggestions. Upon revisiting our previous abstract, we identified several shortcomings. We have made detailed revisions to the abstract based on your feedback, which you can find in the latest manuscript.

4. Keywords: Please list as many keywords as allowed by the journal, chosen from Medical Subject Headings (MeSH) [9], and use as many keywords as possible in the title and in the first two sentences of the abstract [7,8].

**Response:** Based on the content of this manuscript, we have searched for additional keywords in the Medical Subject Headings and incorporated them into the title and abstract wherever possible.

5. Introduction: The authors need to fully expand this section with several paragraphs made up of about 1000 words, introducing information on the main constructs of this protocol, which should be understood by a reader in any discipline, and making it persuasive enough to put forward the main purpose of the current research the authors have conducted and the specific purpose the authors have intended by this study. I would like to encourage the authors to present the introduction starting with the general background, proceeding to the specific background, and finally, the current issue addressed to this study, leading to the objectives. These main structures should be organized in a logical and cohesive manner [10]. In this regard, the manuscript would greatly benefit from incorporating a discussion of the following topics. The following studies, but not limited to, may enhance the value of this manuscript [11–17].

**Response:** Dear Reviewer, thank you for your valuable feedback. We have meticulously revised the introduction based on your suggestions. We carefully reviewed the literature you recommended as well as some additional sources, significantly expanding this section. We have organized our writing more effectively and included citations from many more references, resulting in a noticeable improvement in the readability and usefulness of this part.

6. Methods: I recommend opening this section with a short introductory paragraph and citing more references to ensure the reliability and integrity of the evidence in the study design that the authors built and the methodology they have decided to apply.

**Response:** Thank you for your feedback. We have adjusted the description in this section to make it more concise and readable. Additionally, we have added citations to appropriate places to ensure the reliability and completeness of the methods we employed.

7. Results: I suggest presenting the independent results section and closing this section with a paragraph that puts the results into a more general context.

**Response:** Thank you for your suggestion. We've made adjustments to this section based on your suggestions.

8. Discussion: I would like the authors to present an independent section without subheadings with several paragraphs of about 1500 words, beginning this section with an introduction and providing a summary of the previous section. Then, I expect the authors to develop arguments clarifying the potential of this study as an extension of the previous work, the implications of the findings, how this study could facilitate future research, the ultimate goal, the challenge, the knowledge and technology necessary to achieve this goal, the statement about this field in general, and the importance of this line of research. It is particularly important to present the limitations, merits, and potential translation of this study into clinical practice [18,19].

**Response:** Thank you very much for your suggestions. We recognize the shortcomings in the previous discussion section. Taking your advice into full consideration, we thoroughly revised this part after reading numerous literature sources. We believe that, under your guidance, the quality of our manuscript has significantly improved.

9. Conclusion: I believe that presenting this section with 150–200 words would benefit from a single paragraph that presents some thoughtful and in-depth considerations by the authors as experts to convey the main message. The authors should make an effort to explain the theoretical implications and translational applications of their research. To understand the significance of this field, I believe it is necessary to discuss theoretical and methodological avenues in need of refinement as well as future research directions.

**Response:** We appreciate your insightful suggestions, and have made detailed revisions to this section according to your feedback.

10. References: Please cite more references. An article like this typically cites more than 60 to 70 references.

Overall, the manuscript contains four figures, two table, and 48 references. I believe that the merit of this manuscript lies in its contribution to the understanding of the causal relationship between GERD and migraine, with depression as a mediator. The study used a robust MR method and a large sample size to provide strong evidence supporting the notion that GERD increases the risk of migraine, while migraine does not have a causal effect on GERD. The study also highlights the pivotal role of depression as a bridge in this relationship, with depression mediating a significant proportion of the effect of GERD on migraine. The findings of this study have important implications for the treatment and management of patients with GERD and migraine, particularly those with comorbid depression. The study suggests that interventions targeting GERD may help reduce the risk of migraine and that addressing depression may be an important component of such interventions. Overall, this study provides valuable insights into the complex interplay between gastrointestinal diseases and brain disorders and may pave the way for personalized treatment approaches in the future. I hope that after careful revision, the manuscript meets the journal’s high standards for publication.

**Response:** Dear reviewer, thank you for your affirmation of our work. We hold the utmost respect for your diligent reviewing efforts. This revision process has been significant for us, particularly in light of the valuable suggestions you provided regarding our manuscript. Your meticulous review suggestions demonstrate your high level of professional competence, an aspect we greatly admire and aspire to emulate. We have endeavored to refine our manuscript according to your recommendations, aiming for higher standards of clarity, readability, accuracy, and scientific rigor. We hope our revisions meet the high standards of both you and the journal. Should you have further suggestions, we look forward to continued communication with you. We aspire for our work to contribute to the understanding of the causal relationships between gastroesophageal reflux disease, migraines, and depression.

**Response to Reviewer #3**

Dear Reviewer #3, we greatly appreciate your efforts in reviewing our manuscript. We have carefully considered all your suggestions. We reviewed our research and manuscript in detail and revised the manuscript as carefully as possible. Below is our response to your comments.

1.I have a concern about the exclusion of other variables, such as smoking, alcohol consumption, and obesity, which could also have an impact on the development of GERD and migraine.

**Response:** Dear reviewer, thank you for your feedback. Regarding this issue, we have indeed taken it into consideration. Therefore, our study is divided into three parts. The first part consists of bidirectional two-sample Mendelian randomization analysis, aiming to explore the causal relationship between gastroesophageal reflux disease (GERD) and migraines. In this part, it is essentially a univariable Mendelian randomization analysis, without detailed exclusion of variables such as smoking, alcohol consumption, and obesity. We simply aim to determine whether there is a causal association between GERD and migraines overall, and the direction of this causal association. Hence, there is no need to worry about excluding other variables that may affect the development of GERD and migraines, as this step can be considered as not having excluded variables. In the second part, which is the multivariable Mendelian randomization analysis, we consider the influence of variables such as smoking, alcohol consumption, and obesity. Here, our aim is to explore whether GERD can independently affect migraines apart from these variables. Specifically, whether GERD with pure genetic susceptibility can also promote the occurrence of migraines. Finally, in the third part, we consider depression as a mediating factor to see if we can link GERD, depression, and migraines. This is because previous studies have shown that GERD increases the risk of depression, and depression increases the risk of migraines. However, currently, no one has provided evidence to form a complete disease development curve for these three diseases.

2.Furthermore, it might be preferred to exclude depression as a variable and focus only on the causal effect of GERD on migraine.

**Response:** Dear reviewer, we greatly appreciate your invaluable suggestions. In accordance with your advice, we have supplemented the multivariable Mendelian randomization section by including depression as an additional variable. Our results indicate that even after excluding depression as a variable, gastroesophageal reflux disease (GERD) still has an impact on migraines. Therefore, depression can be considered as one pathway through which GERD promotes the occurrence of migraines, but not the sole factor. This is an intriguing finding. We are sincerely grateful for your thorough review of our manuscript, and we hope that our revisions meet the high standards of both you and the journal.
